# Effective vitrification and warming of porcine embryos using a pH-stable, chemically defined medium

**DOI:** 10.1038/srep33915

**Published:** 2016-09-26

**Authors:** Cristina Cuello, Cristina A. Martinez, Alicia Nohalez, Inmaculada Parrilla, Jordi Roca, Maria A. Gil, Emilio A. Martinez

**Affiliations:** 1 Department of Animal Medicine and Surgery, Faculty of Veterinary Medicine, University of Murcia, E-3100, Murcia, Spain

## Abstract

The use of pH-stable media would simplify embryo vitrification and the warming of porcine embryos and might facilitate the application of embryo transfer in practice. In this work, we investigated whether a pH-stable basal medium constituted of Tyrode’s lactate medium, polyvinyl alcohol, and HEPES for buffering was suitable for porcine embryo vitrification warming in place of the conventional gas-equilibrated media. A high percentage (>90%) of embryos survived vitrification and warming in this medium, achieving *in vitro* survival rates similar to embryos vitrified-warmed using the conventional protocol and their fresh counterparts. The pH-stable medium did not affect the *in vivo* developmental competence of the vitrified-warmed embryos. A farrowing rate of 71.4% (5/7) with 10.4 ± 3.1 piglets born was obtained for the embryos vitrified and warmed in this medium and transferred to selected recipients. This medium will enable the use of simple, safe and standardized protocols for the vitrification and warming of porcine embryos for optimal embryo survival and quality when applied under field conditions. This study opens new possibilities for the widespread use of embryo transfer in pigs.

At present, there is an increasing demand for efficient porcine embryo cryopreservation protocols for industrial and research purposes. Efficient cryopreservation methods would enable the widespread application of porcine embryo transfer programs. These programs allow the global exchange of valuable genetic resources with a minimal risk of disease transmission and a reduced cost, thereby avoiding impacts to animal welfare from transportation^1^,^2^. Unlike the bovine industry, in which vitrification is routinely used in embryo transfer programs and chemically defined media that maintain a stable pH even at ambient temperatures are commercially available, this technology is still in progress in porcines. To date, no reports have described the use of a chemically defined medium with a stable pH as the base medium for porcine embryo vitrification.

Over the last fifteen years, significant advances have been made towards the long-term storage of swine embryos through the development of ultra-rapid vitrification systems^3^. Today, we know a great deal about the vitrification-warming effects on pig embryo viability and quality^4^,^5^,^6^. We also have a great deal of evidence concerning the factors that influence the efficiency of vitrification, such as the embryo developmental stage^7^,^8^, cryoprotectant concentration^9^,^10^ and embryo origin^11^,^12^. This information has revealed the potential for vitrification in swine and is of great value for the selection of the optimal vitrification conditions.

However, for the commercial use of this technology, practitioners demand simple, safe and standardized protocols that warrant optimal embryo survival and quality when applied under field conditions. In this sense, important progress has been made in recent years with the development of a one-step warming procedure^7^ that is useful for direct embryo transfer (ET) or the combined application of vitrification and the non-surgical deep-intrauterine ET procedure^13^,^14^,^15^ developed by our laboratory in the early 2000s^16^,^17^. Recently, the development of chemically-defined vitrification-warming media for *in vivo*^18^ and *in vitro*-derived^12^ embryos has improved the reproducibility of results and reduced the risk of disease transmission associated with animal origin compounds. These vitrification-warming basal media utilize a bicarbonate/CO_2_ buffer system. The inclusion of sodium bicarbonate requires the use of a gassing incubator to maintain the physiological pH (7.2–7.4). Thus, plates containing the vitrification media must be equilibrated in the incubator prior to use. During manipulation, the plates are outside of the incubator, which produces pH changes; therefore, the vitrification and warming plates must be re-equilibrated during the experiments. This process is inconvenient because the gassing incubators are expensive and usually unavailable under field conditions. From a practical and commercial standpoint, it would be preferable to use media with a stable pH that do not require CO_2_ incubators. Additionally, the use of a single medium for the different steps of the technology (embryo collection, vitrification, warming and transfer) would simplify the process and enhance the traceability of the vitrified embryos.

Overall, the aim of this study was to investigate whether Tyrode’s lactate (TL)-HEPES-polyvinyl alcohol (PVA) medium (TL-PVA), which is routinely used in our laboratory for embryo collection and transfer, was suitable as a basal medium (BM) for porcine embryo vitrification and warming. TL-PVA does not require a CO_2_ atmosphere because it does not include a bicarbonate/CO_2_ buffer system, which would simplify the equipment needed for these procedures.

## Results

### Embryo collection

For this study, we selected 58 donor sows that were superovulated and artificially inseminated to collect embryos. On day 6 (day 0 = onset of estrus), 55 out of 58 donors (94.8%) had embryos. The mean ovulation rate in the donor sows was 21.2 ± 3.6 corpora lutea (range 13 to 26), and the recovery rate was 91.8%. Of the recovered structures, 95.2% were embryos, and the rest were unfertilized oocytes and/or degenerated embryos. The total number of embryos collected was 1020, of which 31.2%, 62.7% and 3.8% were morulae, blastocysts and hatched blastocysts, respectively. A total of 939 vitrificable embryos were used in this study. Of these embryos, 589 were used for the evaluation of *in vitro* development (Experiment 1), and the remaining 350 embryos were used for ET (Experiment 2). The rest of the embryos were non-vitrificable embryos due to the presence of hatched blastocysts and unhatched embryos that showed abnormal morphology and therefore were discarded.

### pH values of the vitrification and warming media based on TCM or TL-PVA

**Figure 1:**
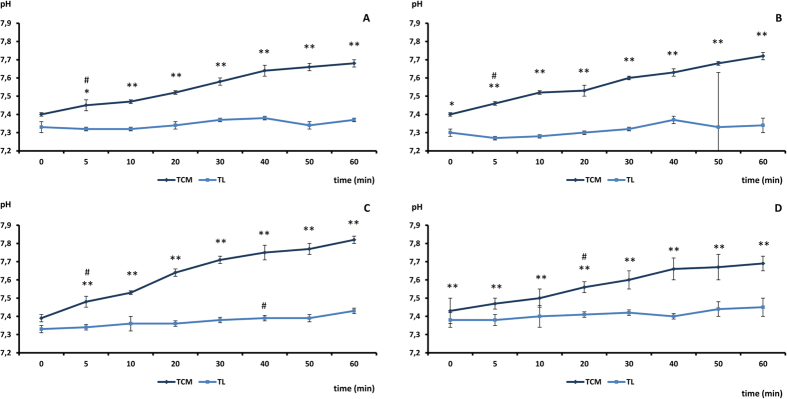
pH values of basal (**A**), warming (**B**) and vitrification media (V1: (**C**) and V2: (**D**)) prepared using TCM or TL-PVA (TL) as the basal medium. Measurements of pH values were performed 0, 5, 10, 20, 30, 40, 50 and 60 min after the vitrification and warming plate preparation. Asterisks indicate significant differences between both media for each time point (*P < 0.05; **P < 0.01). A hash mark (#) indicates that from this time onwards the pH values significantly (P < 0.05) differed from the initial pH.

We performed a preliminary test to determine the pH variations in the vitrification and warming media prepared using either TCM or TL-PVA as the basic medium and maintained at 39 °C without re-equilibration in the CO_2_ incubator during manipulations. The pH of the media was recorded 0, 5, 10, 20, 30, 40, 50 and 60 min after preparation of the vitrification and warming plates. Three replicates of this test were performed. The BM had a significant effect (P < 0.01) on the pH values of the vitrification and warming media maintained at 39 °C outside of the incubator (Fig. 1). The TCM and TL-PVA media displayed a pH between 7.35 and 7.40 at time 0. After 5 min, the TCM-based media displayed a higher (P < 0.05) pH than their counterparts in every replicate until the end of the measurements. The TCM-based media suffered a more marked (P < 0.01) alkalinization than the media based on TL-PVA, with overall pH increases of 0.33 ± 0.08 and 0.09 ± 0.05, respectively. The TL-PVA media displayed a stable pH; only the final pH of TL-PVA-V2 and of TL-PVA-V1 from the 40^th^ to the 60^th^ min differed (P < 0.05) from the initial pH value. Due to the pH instability of the TCM medium outside of the CO_2_ incubator without re-equilibration, which resulted in a pH outside of the optimal range for pig embryos (7.2–7.4), the vitrification and warming protocols using TL-PVA-based media were compared only with the conventional vitrification and warming protocols using TCM equilibrated before and during the manipulations in experiments 1 and 2.

### *In vitro* embryo development after vitrification and warming using TL-Hepes or TCM-based medium

In this first experiment, we examined the *in vitro* embryo development of morulae and blastocysts vitrified-warmed using either the conventional procedure with equilibrated-TCM medium or the TL-Hepes-based medium. Morulae and blastocysts were collected from 33 donor sows in 3 replicates and pooled according to their developmental stages. Some embryos were cultured *in vitro* without vitrification to evaluate the *in vitro* development capacity of the fresh blastocysts (n = 137). The remaining embryos (n = 452) were equally and randomly allocated to one of three experimental groups as follows: 1) TCM-TCM, in which the embryos were vitrified and warmed using equilibrated TCM as the BM; 2) TL-TCM, in which the embryos were vitrified using TL-PVA and warmed using equilibrated TCM as the BM; and 3) TL-TL, in which the embryos were vitrified and warmed using TL-PVA as the BM. Fresh and vitrified-warmed morulae and blastocysts were cultured *in vitro* for 48 and 72 h, respectively. The embryo survival and hatching rates were evaluated during culture. At the end of the embryo culture, the total blastocyst cell number was assessed.

**Figure 2:**
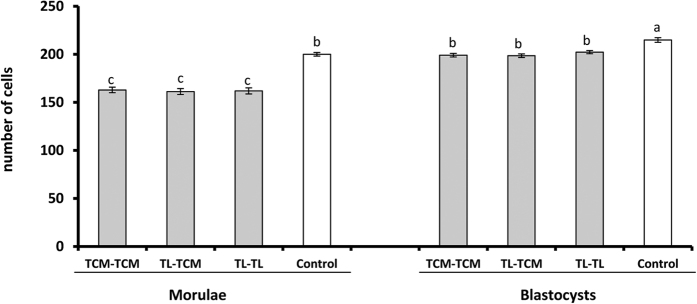
Total cell numbers in blastocysts derived from fresh (white bars) and vitrified morula and blastocysts (grey bars) after 48 and 72 h of *in vitro* culture, respectively. The embryos were vitrified-warmed using TCM as the basic medium (TCM-TCM), vitrified using TL-PVA as basic medium and warmed with TCM (TL-TCM) or vitrified and warmed using TL-PVA (TL-TL). Data are expressed as the mean ± SD. Different letters represent differences (P < 0.05) among groups.

The basal media used for vitrification and warming had no effect on the *in vitro* developmental competence of the vitrified-warmed embryos (Table 1). A high percentage (>90%) of embryos survived vitrification and warming, achieving survival rates similar to those of their fresh counterparts (Table 1). The hatching rate was affected by the embryo developmental stage (P < 0.01) and its interaction with the vitrification-warming group. The vitrified morulae hatched *in vitro* at a lower (P < 0.01) proportion than the vitrified blastocysts and fresh embryos. There was no significant difference in the total cell numbers between the vitrification-warming groups within the same embryo developmental stage (Fig. 2). However, the embryo developmental stage had a significant (P < 0.05) effect on this parameter. Thus, the blastocysts derived from morulae had a lower (P < 0.05) total cell number than those derived from blastocysts. Vitrification also affected the total cell number, with the vitrified embryos having a lower (P < 0.05) total cell number than their fresh equivalents (Fig. 2).

### *In vivo* embryo development of blastocysts vitrified-warmed using conventional TCM or TL-Hepes-based medium

Next, we evaluated the reproductive parameters obtained after the transfer of blastocysts vitrified-warmed with the conventional protocol or with the new TL-based media, which did not require gassing equilibration to maintain a stable pH. For this experiment, a total of 350 blastocysts were collected from 22 donor sows. Blastocysts from each donor were equally and randomly allocated into the following experimental groups: the TCM group, in which the embryos were vitrified and warmed using equilibrated TCM as the BM, and the TL group, in which the embryos were vitrified and warmed using TL-PVA as the BM. The vitrified-warmed embryos were transferred to a total of 14 recipient sows (7 per group). The recipients were checked for estrus signs beginning 12 days after ET. Pregnancy was diagnosed by ultrasonography on days 20 to 22 post-transfer. Pregnant sows were allowed to carry the litters to term. The variables recorded were the pregnancy rate, litter size, number of born alive piglets and piglets’ birth weights. The *in vivo* embryo survival was calculated as the proportion of live-born piglets with respect to the number of embryos transferred to all sows and to the farrowed sows. The overall piglet production efficiency was defined as the ratio of the number of piglets born to the number of embryos transferred to all recipients. A regular return to estrus (19–23 days) was observed in three recipients, and the remaining eleven recipients were diagnosed as pregnant by ultrasonography (Table 2). One pregnant recipient from the TL group returned to estrus on day 35. The remaining ten recipients came to term (overall 71.4% farrowing rate) and farrowed an average of 10.2 ± 2.3 piglets (range from 7 to 14), of which 9.8 ± 1.8 were born alive. The average weight of the piglets at birth was also similar for the TCM and TL groups (1.47 ± 0.3 kg and 1.65 ± 0.3 kg, respectively). No morphological abnormalities were observed in the piglets. Altogether, the *in vivo* embryo survival for both vitrification groups was 28.0% overall and 39.2% in the sows that farrowed. The overall piglet production efficiency was 29.1%.

## Discussion

This study is the first report of the use of a single chemically-defined medium that does not require a CO_2_ atmosphere to maintain a stable pH for embryo collection, vitrification, warming and transfer in swine. The results obtained in this study with the TL-PVA medium buffered with HEPES might be useful for the practical application of these technologies. Although several buffers have been used for mammalian embryos, recent results obtained in bovines^19^ indicated that HEPES-buffered media were highly efficient for the storage of embryos, leading to a highly stable pH and the preservation of embryo viability.

An acceptable pH range for embryo culture media may be set between pH 7.2 and 7.4^20^. Our conventional vitrification-warming protocol includes TCM-based media, which must be equilibrated before and during manipulation. Under these conditions, the pH values of the TCM-based media are stable and allow very good results to be obtained, as revealed by our previous *in vitro*^18^,^21^ and *in vivo*^14^,^15^ studies. However, with the goal of simplifying vitrification and to develop warming procedures that are useful under field conditions, the use of CO_2_ incubators during the process should be avoided. The results from our preliminary experiment indicate that the initial pH of the TCM-based media is in an optimal range for embryos (from 7.3 to 7.4^22^,^23^) after gassing equilibration. Nevertheless, without re-equilibration throughout the manipulations, the pH increases, achieving a final rise of 0.33 ± 0.08 points corresponding to final pH values that can compromise embryo development and quality^24^,^25^,^26^. The logarithmic pH scale can give the false impression of minor pH changes, but we should consider that a change in pH of one corresponds to a 10-fold change in the H^+^ concentration. Raising the pH over 7.4 has been reported to cause disruption of mitochondria and actin microfilaments in mouse embryos^27^. Additionally, increasing the pH by only approximately 0.1–0.15 units over a 7.4 value has been proven to affect embryo metabolism by activating glycolysis and lowering the oxidative metabolism in mouse^28^ and hamster^29^ embryos. Another concern regarding pH is that vitrification reduces the ability of the embryo to regulate its intracellular pH for a period of at least 6 h^29^. As a consequence, these embryos are more sensitive to pH oscillations. On this subject, an adequate pH of the warming medium should be essential for minimizing the stresses imposed on vitrified embryos during manipulations before transfer. Considering this evidence and the pH oscillations observed in the TCM-based media, we recommend that TCM-based media not be used without re-equilibration in a CO_2_ incubator. Unlike the TCM medium, the medium based on TL-PVA displayed very stable pH values. Only the pH values at 60 min were significantly (P < 0.01) higher than the initial pH values, although they remained within the optimal pH range (7.2–7.4), suggesting that this increase was safe for embryos. The TL-PVA medium, which is routinely used in our laboratory for embryo collection and warming, seems to be a good candidate BM for vitrification and warming under field conditions. The pH values obtained with the TCM-based media contrast with the results reported by Ideta *et al*.^19^, who preserved bovine embryos at 4 °C for a week in TCM 199 supplemented with 25 mM HEPES and 50% fetal bovine serum. In that study, the embryo viability was preserved, indicating that the pH of the medium remained in an optimal range for embryos during storage. The medium used by Ideta *et al*. was quite similar to the TCM used in this study; however, in that study, storage was performed in a sealed straw with the medium containing the embryos avoiding any contact with the air and no manipulations were performed during storage.

In Experiment 1, the embryos were vitrified using the conventional protocol with TCM (equilibrated before use and throughout the manipulations) and achieved very high *in vitro* survival and hatching rates, which were similar to those achieved previously using analogous conditions^8^,^14^,^18^. When TL-PVA was used as the BM for vitrification and warming, the *in vitro* embryo survival rate was comparable to the rate obtained with the conventional protocol. Additionally, the embryo quality based on the total number of cells in the blastocyst was not affected. These observations support the claim that TL-PVA can substitute for TCM in vitrification and warming protocols without altering the *in vitro* outcomes of the morulae and blastocysts. Our results revealed an effect of the embryo developmental stage, with morulae showing a lower hatching rate than their fresh counterparts and blastocysts, which was consistent with earlier studies^7^,^8^. Published studies to date have considered the embryo developmental stage to be one of the main factors affecting vitrification^4^,^9^,^30^. Although morulae tend to have a reduced vitrification ability compared to blastocysts, a recent study found that these differences were not evident after the non-surgical transfer of porcine embryos vitrified using our conventional protocol^15^. This observation led us to assume that vitrified-warmed morulae probably hatched at a higher proportion *in vivo* than under *in vitro* conditions. The reason for these differences is unclear, but they may be related to the detrimental effects of the *in vitro* culture environment on embryos^31^,^32^,^33^. In addition to the embryo developmental stage, other important factors could affect the vitrification efficiency, such as the type of cryoprotectant, their concentrations, the vitrification container or the cooling rate; these factors should be taken into account for the development of efficient vitrification-warming protocols.

The results from Experiment 2 demonstrated that a good reproductive performance could be achieved after the surgical transfer of 25 porcine blastocysts vitrified and warmed using TL-PVA as the BM, with results comparable to those obtained with the conventional medium. The overall piglet production efficiency (29.1%) obtained in this study was higher than the efficiency reported in the pioneering studies in this field^34^ in which only 20 vitrified/warmed embryos were transferred (range 6% to 14.5%). The improvement in results might be a consequence of different ameliorations in the vitrification and warming procedures, such as the reduction in the cryoprotectant concentration^10^ and the number of embryos transferred. In this respect, studies transferring only 20 embryos^34^,^35^ have reported a lower piglet production efficiency (range 6% to 13.0%) than those transferring 25 to 30 embryos (range 17.2% to 23.4%)^15^,^36^,^37^,^38^. The reproductive performance obtained in the present study is comparable to that reported recently by our group^15^ for the transfer of 30 embryos vitrified with our conventional protocol in which an overall pregnancy rate of 76.5% and an overall piglet production efficiency of 23.4% were achieved. In that study, we observed that the surgical ET procedure required a lower number of vitrified-warmed embryos compared with non-surgical ET to achieve a comparable efficiency^15^. This difference could be due to the placement of the embryos transferred, because the surgical procedure allows for the deposition of blastocysts in the tip of the uterine horn, which is their physiological location on day 6^39^. Using this advantage, we have reduced the number of transferred embryos from 30 to 25 by maintaining the reproductive parameters obtained in that study. Based on our own experience and the findings of previous reports^15^,^36^,^37^,^38^, we propose that the proper number of vitrified morulae and blastocysts needed for surgical transfer in swine is between 25 and 30. However, factors such as *in vitro* embryo survival must be considered before establishing this parameter.

In conclusion, our study demonstrates that vitrification and warming procedures can be performed using TL-PVA as a BM, thereby ensuring an excellent *in vitro* developmental potential of the vitrified morulae and blastocysts and an adequate reproductive performance after surgical embryo transfer. The use of media that do not require gassing equilibration will facilitate the application of porcine embryo vitrification and ET in practice.

## Methods

### Chemicals

The chemicals used in this study were purchased from Sigma-Aldrich Quimica S.A. (Madrid, Spain) unless otherwise indicated.

### Animals

This work was conducted under field conditions in a commercial farm in southeastern Spain (Agropor SA, Murcia, Spain). Crossbred sows (Landrace × Large-White) from the same genetic line (from two to six parities) were used as embryo donors and recipients. The sows were located individually in crates in a mechanically ventilated confinement facility and fed a commercial ration twice a day. Water was provided *ad libitum*. This study was performed in accordance with Directive 2010/63/EU EEC for animal experiments, and all experiments were evaluated and approved in advance by the Bioethical Committee of the University of Murcia, Spain (research code: 638/2012).

### Detection of estrus and insemination

Estrous detection was conducted once a day (at 7:00 a.m.) beginning 2 days after weaning by allowing snout-to-snout contact of females with vasectomized mature boars and by applying manual back pressure. Females that showed the standing estrus reflex were considered to be in estrus. Only sows with a weaning-to-estrus interval of 4 to 5 days were selected as donors or recipients. Donors were post-cervically inseminated 6 and 24 h after the onset of estrus with 1.5 × 10^9^ spermatozoa in 45 mL doses prepared with semen from two adult Pietrain boars extended with BTS (“Beltsville Thawing solution”)^40^ and stored at 17 °C for a maximum of 24 h.

### Embryo collection

Embryo collection was performed in a surgical room located on the farm. Morulae and blastocysts were collected by mid-ventral laparotomy on day 6 of the estrous cycle (D0: onset of estrus). The donors were sedated using azaperone (2 mg/kg body weight, i.m.). General anesthesia was induced with sodium thiopental (7 mg/kg body weight, i.v.) and maintained with isoflurane (3.5–5%). The reproductive tract was exposed via a mid-line incision, and the corpora lutea in the ovaries were counted. Embryos were collected by washing the tip of each uterine horn with 30 mL of TL-PVA^41^ with some modifications^42^. This medium contains 0.1 g/L PVA, 10 mM HEPES and 2 mM NaHCO_3_. The TL-PVA used for flushing was also used for vitrification-warming and embryo transfer. After flushing, the recovered embryos were evaluated under a stereomicroscope at a 60× magnification to verify their developmental stage and quality. One-cell eggs and poorly developed embryos were classified as unfertilized oocytes and degenerated embryos, respectively. Only morulae and blastocysts with intact zona pellucida showing good or excellent morphological appearances^43^ were selected for the experiments. The collected embryos were washed three times in TL-PVA, placed in Eppendorf tubes containing 1.5 mL of the same medium and transported to the laboratory in a portable incubator set at 39 °C within 2 h after collection.

### Vitrification and warming

**Figure 3:**
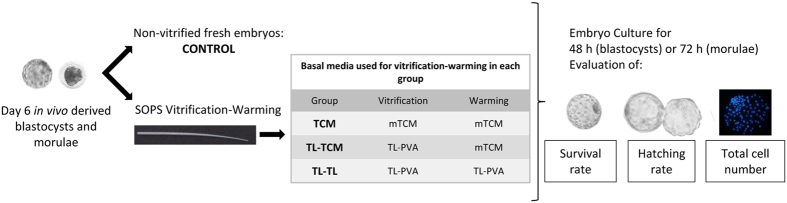
Experimental design of experiment 1 (*In vitro* embryo development after vitrification and warming using TL-Hepes or TCM-based media).

**Figure 4:**
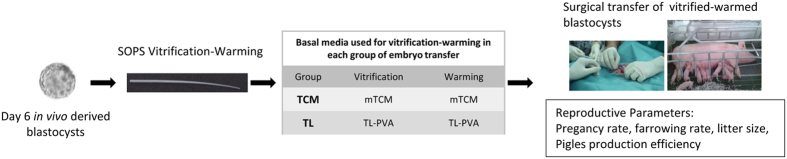
Experimental design of experiment 2 (*In vivo* embryo development of blastocysts vitrified-warmed using conventional TCM or TL-Hepes-based media).

The embryos were washed four times in TL-PVA at 39 °C. Vitrification was performed within 3 h after embryo collection. The morulae and blastocysts were vitrified using the method described by Berthelot *et al*.^34^ and modified by Cuello *et al*.^10^. The BM for vitrification was either TCM199 (M7528) containing 25 mM HEPES and 26.19 mM sodium bicarbonate and supplemented with 0.5 mM L-glutamine and 0.1% PVA (TCM) or TL-PVA, which was also used for the embryo collection and transfer. All media were maintained at 39 °C. The media based on TCM were equilibrated in a CO_2_ incubator for 2 h before use. Prior to vitrification, the embryos were washed twice in BM. Then, groups of five to seven embryos were sequentially equilibrated in the first vitrification medium (V1: BM + 7.5% (v/v) dimethyl-sulfoxide + 7.5% (v/v) ethylene glycol) for 3 min and in the second vitrification medium (V2: BM + 16% (v/v) dimethyl-sulfoxide + 16% (v/v) ethylene glycol + 0.4 M sucrose) for 1 min. During the last step, the embryos were placed in a 1 μL drop and loaded into the narrow end of a Superfine Open Pulled Straw (SOPS, Minitüb, Tiefenbach, Germany) by capillary action. Subsequently, the straws containing the embryos were plunged horizontally into liquid nitrogen (LN_2_). The vitrified embryos were stored in LN_2_ for 2 to 3 weeks. After storage in LN_2_, the embryos were warmed by the one-step dilution method^5^,^8^. A warming medium was prepared using either TCM or TL-PVA according to the experimental design. After warming, the embryos were cultured *in vitro* (Experiment 1; Fig. 3) or transferred to recipient sows (Experiment 2; Fig. 4).

### *In vitro* embryo culture and assessment of *in vitro* embryo development and total blastocyst cell numbers

**Figure 5:**
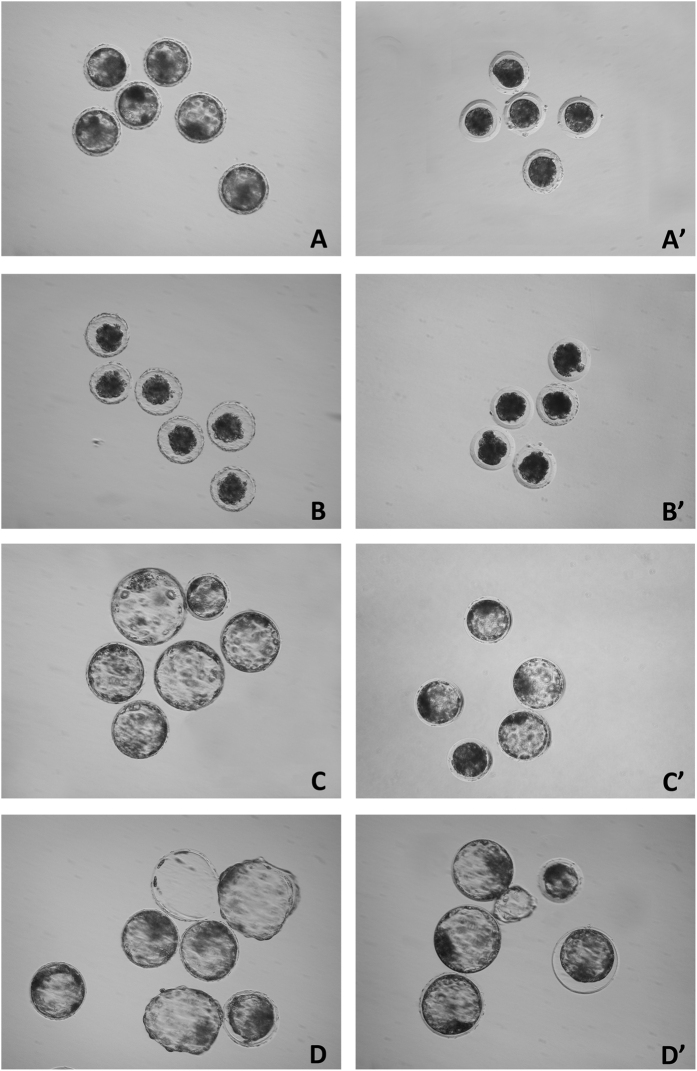
Stereomicroscopic images of fresh and vitrified–warmed blastocysts (**A–D**) and morulae (**A’–D’**). (**A,A’**) Fresh blastocysts and morulae before vitrification. (**B,B’**) Blastocysts and morulae immediately after warming. (**C,C’**) Vitrified-warmed blastocysts and morulae after 24 h of *in vitro* culture. (**D,D’**) Vitrified-warmed blastocysts and morulae after 48 h of *in vitro* culture.

To assess the survival and hatching rates of vitrified-warmed embryos *in vitro*, the morulae and blastocysts were cultured for 48 h and 72 h, respectively, in four-well multidish plates containing 500 μL of NCSU-23^44^ droplets supplemented with 0.4% bovine serum albumin and 10% fetal calf serum at 39 °C in 5% CO_2_ in air. During the culture period, the embryos were morphologically evaluated for their developmental progression by stereomicroscopy (Fig. 5). The vitrified/warmed morulae that progressed to the blastocyst stage during culture and the vitrified blastocysts that reformed their blastocoelic cavities after warming were considered viable if they displayed a normal or thinning zona pellucida with an excellent or good appearance during the culture (Fig. 5). The survival rate was defined as the ratio of viable embryos to the total number of embryos cultured. The hatching rate was calculated as the ratio of the number of hatched embryos *in vitro* to the total number of embryos cultured. The hatched blastocysts were fixed in 4% paraformaldehyde in PBS for 30 min at room temperature (22–24 °C). Then, the blastocysts were washed with 3 mg/mL of BSA, placed on a slide in 4 μL of Vectashield (Vector, Burlingame, CA, USA) containing 10 μg/mL of Hoeschst 33342 and covered with a coverslip. The fixed embryos were examined with a fluorescence microscope using a 330–380 nm excitation filter. The total number of cells with nuclei that displayed blue fluorescence was counted. All cell counts were performed by the same person.

### Embryo transfer

The embryos were transferred surgically by mid-ventral laparotomy in asynchronous (the onset of natural estrus appeared 24 h after that of the donors) recipients. One h before the transfer, each recipient received a dose of 15 mg/kg of a long-acting amoxicillin suspension (Clamoxyl LA, Pfizer, Madrid, Spain) via intramuscular injection. The recipients were sedated and anesthetized as described previously for embryo collection. Immediately after warming, the embryos (N = 25) were loaded into a Gynétics embryo transfer catheter (Gynétics Medical Products N.V., Lomel, Belgium) connected to a 1 mL syringe. The ET catheter was loaded with air bubbles that separated the drop containing the embryos (maximum volume of 30 μL of TL-PVA) from two drops of TL-PVA before and after the drop containing the embryos. The embryos were transferred into the tip of a uterine horn (5–6 cm from the utero-tubal junction) by inserting the Gynétics catheter through the uterine wall, which was previously punctured with a blunt Adson tissue forceps. Forty-eight h after ET, another 15 mg/kg dose of a long-acting amoxicillin suspension was administered to each recipient.

### Statistical analysis

The data were analyzed using IBM SPSS 19 Statistics (SPSS, Chicago, IL, USA). The pH values (preliminary pH test) and total piglets born and born alive (Experiment 2) were compared by an unpaired Student t-test corrected for inequality of variances (Levene test). The mean ± standard deviation (SD) of the binary variables (survival and hatching rates) from Experiment 1 was obtained after calculating the percentage in every group and in each replicate. The variables were evaluated using the Kolmogorov-Smirnov test to assess the assumption of normality, and the groups were compared using a mixed-model ANOVA. The ANOVA model included the fixed effects of the vitrification-warming group, the embryo developmental stage and their interactions and the random effects of the replicate. When the ANOVA revealed a significant effect, the values were compared using Bonferroni’s test. In Experiment 2, the percentage data (pregnancy and farrowing rates and *in vivo* embryo survival and piglet production efficiency) were compared using Fisher’s exact test. Differences were considered significant when P < 0.05. The results are expressed as percentages and means ± SD.

## Additional Information

**How to cite this article**: Cuello, C. *et al*. Effective vitrification and warming of porcine embryos using a pH-stable, chemically defined medium. *Sci. Rep.* **6**, 33915; doi: 10.1038/srep33915 (2016).

## Figures and Tables

**Table 1:** *In vitro* embryo survival and hatching rates of porcine morulae and blastocysts vitrified and warmed using TL-PVA and/or TCM as the basal medium.

Embryo stage	Vitrification BM	Warming BM	Number of embryos (N)	Survival rate (%)	Hatching rate (%)
Morulae	TCM	TCM	82	95.3 ± 3.7	51.1 ± 3.1^a^
	TL	TCM	80	90.2 ± 5.2	55.2 ± 4.9^a^
	TL	TL	76	94.8 ± 4.3	56.6 ± 4.6^a^
	Fresh control		70	98.6 ± 2.8	94.3 ± 0.2^b^
Blastocysts	TCM	TCM	72	97.1 ± 3.3	94.4 ± 4.5^b^
	TL	TCM	70	95.7 ± 2.9	92.8 ± 3.1^b^
	TL	TL	72	95.9 ± 2.7	90.3 ± 2.5^b^
	Fresh control		67	100 ± 0.0	98.1 ± 3.8^b^

BM: Basal medium; TCM: modified TCM-199; TL: TL-PVA. Dissimilar letters in the same column indicate significant differences (P < 0.01). Data are presented as the mean ± SD (three replicates).

**Table 2:** Reproductive variables of recipients after the surgical transfer of 25 vitrified-warmed blastocysts.

Experimental group	TCM	TL
Recipients (N)	7	7
Blastocysts transferred per recipient	25	25
Pregnancy (25 d), N (%)	5 (71.4)	6 (85.7)
Farrowing, N (%)	5 (71.4)	5 (71.4)
Total born (mean ± SD)	10 ± 1.6	10.4 ± 3.1
Born alive (mean ± SD)	9.6 ± 1.1	10.0 ± 2.6
Overall piglet production efficiency* (%)	28.6	29.7

**TCM**: Blastocysts were vitrified and warmed using gassed equilibrated TCM as a basal medium. **TL**: Blastocysts were vitrified and warmed using TL-PVA as a basal medium. *The overall piglet production efficiency was calculated as the ratio of the number of total piglets born to the number of embryos transferred to all recipients.
